# Handling of antineoplastic drugs: a health concern among health care workers

**DOI:** 10.47626/1679-4435-2020-527

**Published:** 2021-03-03

**Authors:** Stephanie Damasceno Rocha, Andre Nascimento Honorato Gomes, Paulo Ricardo Gazzola Zen, Claudia Giuliano Bica

**Affiliations:** 1 Departamento de Ciências Básicas da Saúde, Universidade Federal de Ciências da Saúde de Porto Alegre, Porto Alegre, RS, Brazil; 2 Escola de Enfermagem de Manaus, Universidade Federal do Amazonas, Manaus, AM, Brazil

**Keywords:** antineoplastic drugs, occupational health, health care providers, occupational risks

## Abstract

**Introduction:**

Health care professionals are part of a group that is more exposed to a wide range of sources of risk that are very harmful to their own health. Antineoplastic drugs are widely used to treat many different types of cancer and are very aggressive to both patients and health care professionals.

**Objectives:**

To identify occupational risks and assess knowledge in health care professionals from Porto Alegre whose work involves handling antineoplastic drugs.

**Methods:**

This was a prospective, descriptive, and cross-sectional study with qualitative and quantitative analyses. It was conducted in two stages. A questionnaire containing objective questions was administered in stage one. In stage two, observations were made during regular visits to the sites studied at different times, following a checklist based on the requirements of health regulation standards relating to handling of antineoplastic drugs.

**Results:**

A total of 40 health care professionals took part in the study, 11 nurses, 14 pharmacists, and 15 nursing and/or pharmacy technicians. Twenty-seven of them had been involved in some type of accident during their professional practice. It was also observed that the institutions were making efforts to comply with legal requirements, since 32 reported that they took part in the Program for Medical Control of Occupational Health and 29 of the employees stated they had had some type of training in the antineoplastic area.

**Conclusions:**

Exposure to antineoplastic drugs through contact, aerosols, ingestion, and inhalation was detected. Additionally, ergonomic, physical, and biological risks were also present, since working with different pathological organisms and working processes impacts on these workers’ health. Assessment of the health care professionals’ knowledge identified a lack of knowledge and weaknesses with relation to handling this class of drugs.

## INTRODUCTION

Health care workers are part of a group that is more exposed to a wide range of sources of risk that are very harmful to their own health.^1^ According to Marziale,^2^ chemical factors stand out among the major risk factors. These substances are used in their different physical states as work tools by health care professionals, both when administrating and when handling medicines. Antineoplastic drugs are one of the most important classes among the chemicals employed in health care.

These drugs are commonly used to treat various types of cancer and are aggressive to those who use them (patients) and to those handling them (health care workers). Several studies have evaluated exposure risks and the effects antineoplastic drugs can have on health workers during administration.^3,4^

The United States’ National Institute for Occupational Safety and Health (NIOSH) has compiled a list of 167 hazardous drugs that can be considered dangerous to those who handle them. More than half of these substances are antineoplastic drugs. It should also be noted that these drugs are potentially mutagenic, carcinogenic, teratogenic, and reprotoxic.^5^ These substances can cause health damage to workers, inducing cancer and bone marrow depression (granulocytopenia, anemia, and thrombocytopenia and changes to the immune system, liver, and fertility, among others).^6,7^

In health care facilities, working with or near these drugs can also lead to skin rashes, infertility, miscarriage, and birth defects, and possibly to development of leukemia or other malignancies.^7^

Side effects that these drugs can generate in workers who handle them have been studied since the advent of antineoplastic drugs and anticancer therapy. Several studies have demonstrated the health risks to the workers who use these drugs.^8-11^

Over the years, various rules, regulations, and guidelines have been proposed to control occupational exposure to cytotoxic drugs. These regulations cover all aspects, including administrative control, engineering control, and personal protective equipment (PPE).^6,7,12^

In Brazil, many International Labor Organization (ILO) conventions and recommendations have been ratified in Ordinances of the Ministry of Labor, known as Regulatory Standards, in addition to Consolidation of Labor Laws covering this area. There are 36 regulatory standards regulated by the Brazilian Ministry of Labor, several of which stand out for their importance to safeguarding the health of workers handling antineoplastic agents: NR-5: Internal Commission for Accident Prevention, NR-6: Personal Protective Equipment (PPE); NR-7: Program for Medical Control of Occupational Health, and, the most important, NR-32: Safety and Health at Healthcare Facilities. These regulations establish basic guidelines for implementation of measures to protect the safety and health of workers in general, as well as those who work in health care.^13-15^

It is unacceptable that, in the twenty-first century, there are still health care workers who are unaware of and do not know the rules to apply in cases of contamination and clinical manifestations caused by this type of substance.

Studies show that there are still many issues related to risk and safety standards among health care workers.^3,11,16,17^ However, to what extent do these professionals actually not know the risks or have they stopped believing in these dangers and are acting recklessly? The health of workers exposed to antineoplastic drugs is a constant and global concern.

Therefore, this study aims to identify the main current occupational hazards faced by health care workers in Porto Alegre who work handling antineoplastic drugs and assess their knowledge on the subject.

## METHODS

This is a prospective, descriptive, cross-sectional study with qualitative and quantitative analyses.

## PARTICIPATING HOSPITALS

The study population comprised health care workers at the three major referral centers for chemotherapy treatment in Porto Alegre, southern Brazil.

All study participants were professionals who worked handling antineoplastic drugs. The sample comprised nurses, pharmacists, and nursing and pharmacy technicians. All students (interns or residents) studying on health care courses or for higher level technical qualifications were excluded.

According to Brazilian regulatory standards, this study could only start after the approval from the institutional ethics committees at each site. All participants agreed to participate and signed informed consent forms before any interventions.

This study was divided into two stages for data collection:

- Stage 1 - Questionnaire: The data collection instrument used was a questionnaire developed and adapted from Brazilian regulatory standards (ANVISA) and available literature on the subjects of biosafety/occupational health/chemotherapeutic agents.^13,18^ This instrument contained 25 objective questions with multiple choice response options covering the topics described above.
- Step 2 - Observation: Periodic visits were made to the chemotherapy referral sites at different times following a checklist of requirements based on regulatory standards on occupational health and safety in handling antineoplastic drugs.^19^

## ANALYTICAL PROCEDURE

A descriptive analysis of the data was performed. All qualitative variables were expressed as percentages and quantitative variables were expressed as means. All data were statistically analyzed using the frequency test and Pearson’s chi-square. Variables with p < 0.05 were considered significant.

## RESULTS

A total of 40 health care workers who worked handling chemotherapy drugs at the three chemotherapy referral sites in Porto Alegre were recruited from January to October of 2013. They were classified into three professional categories: 27.5% (11) nurses, 35% (14) pharmacists and 37.5% (15) technicians. Fourteen (35%) of them were between 30 to 40 years old. It can be observed from the demographic data shown in Table 1 that many women (90% [36]) were enrolled on this study and just 10% (4) of the sample were men.

**Table 1:** Description of demographic data for the study population, Porto Alegre, Brazil, 2014

Demographic variables	Nurse n (%)	Pharmacist n (%)	Technician n (%)	Total n (%)
Gender				
Male	0 (0.0)	3 (75.0)	1 (25.0)	4 (10.0)
Female	11 (30.5)	11 (30.5)	14 (38.8)	36 (90.0)
Age (years)				
< 20	0 (0.0)	0 (0.0)	0 (0.0)	0 (0.0)
20-30	1 (8.3)	6 (50.0)	5 (41.6)	12 (30.0)
30-40	4 (28.5)	7(50.0)	3 (21.4)	14 (35.0)
> 40	6 (46.1)	1 (7.69)	6 (46.1)	13 (32.5)
Did not answer	0 (0.0)	0 (0.0)	1 (100.0)	1 (2.5)
Working hours				
4	0 (0.0)	2 (50.0)	2 (50.0)	4 (10.0)
6	6 (85.7)	6 (31.5)	7 (36.8)	19 (47.5)
8	5 (83.3)	6 (35.3)	6 (35.2)	17 (42.5)
> 12	0 (0.0)	0 (0.0)	0 (0.0)	0 (0.0)
Shift				
One	8 (34.8)	7 (30.4)	8 (34.8)	23 (57.5)
Two	3 (17.6)	7 (41.2)	7 (41.2)	17 (42.5)
Work experience (years)				
< 1	0 (0.0)	1 (16.6)	5 (83.3)	6 (15.0)
1-5	3 (17.6)	8 (47.0)	6 (35.3)	17 (42.5)
6-15	4 (36.3)	4 (36.3)	3 (27.3)	11 (27.5)
> 15	4 (66.6)	1 (16.6)	1 (16.6)	6 (15.0)
Program for Medical Control of Occupational Health				
Yes	10 (27.0)	14 (37.8)	13 (35.1)	37 (92.5)
No	1 (33.3)	0 (0.0)	2 (66.6)	3 (7.5)
Training				
Yes	7 (24.1)	12 (41.3)	10 (34.5)	29 (72.5)
No	4 (36.3)	2 (18.2)	5 (45.4)	11 (27.5)

Risks of accidents at work and occupational diseases can be attributed to risk factors, including ergonomic, psychosocial, chemical, physical, and biological risk factors, which can compromise productivity, the quality of care provided and workers’ health. Ergonomic risks are those factors that can affect workers’ physical or mental integrity, causing discomfort or illness, which can generate physiological and psychological disorders. Psychosocial risks are caused by the nursing professionals’ greater contact with their patients’ suffering; physical factors can include noise, vibrations, ionizing or other types of radiation, and infrasound or ultrasound. Biological factors are caused by pathogenic microorganisms; and chemical factors comprise chemicals that can penetrate the body through contact with the skin or airways or if swallowed.

Among health care workers who reported having suffered from some sort of exposure to neoplastic drugs, 63% (17) were only working on one shift. Almost all workers, 92.5% (32), reported participating in the Program for Medical Control of Occupational Health and more than half of them (72.5% [29]) reported having had some training in the area.

Investigating the rate of exposure (Table 2), 67.5% (27) of participants reported having suffered some sort of accident related to neoplastic drugs. Analysis of the number of accidents reported, taking into account occupation categories and the total number of victims revealed that pharmacists were the class of workers who reported most exposure (40.74% [11]), followed by nurses 33.33% (9), and technicians 25.92% (7). When asked whether they had submitted a Work Accident Statement, only 44.4% (12) of the 27 health care workers who reported having had some type of accident said they had made a statement, while the remaining 51.9% (14) had not done so.

**Table 2:** Exposure to antineoplastic drugs at work, Porto Alegre, Brazil, 2014

	Nurses n (%)	Pharmacists n (%)	Technicians n (%)	Total n (%)
Exposure				
Yes	9 (33.3)	11 (40.7)	7 (25.9)	27 (67.5)
No	2 (15.4)	3 (23.1)	8 (61.5)	13 (32.5)
Number of exposure events				
Less than 5	5 (33.3)	7 (46.7)	3 (20.0)	15 (55.6)
5 to 15	3 (30.0)	3 (30.0)	4 (40.0)	10 (37.0)
More than 15	1 (50.0)	1 (50.0)	0 (0.0)	2 (7.4)
Type of exposure				
Aerosols	1 (25.0)	2 (50.0)	1 (25.0)	4 (14.8)
Contact	9 (34.6)	11 (42.3)	6 (23.1)	26 (96.3)
Ingestion	0 (0.0)	0 (0.0)	1 (100.0)	1 (3.7)
Inhalation of medication	0 (0.0)	1 (100)	0 (0.0)	1 (3.7)
Symptom				
Yes	3 (37.5)	3 (37.5)	2 (25.0)	8 (29.6)
No	6 (31.6)	8 (42.1)	5 (26.3)	19 (70.4)
Work accident statement				
Yes	2 (16.7)	6 (50.0)	4 (33.3)	12 (44.4)
No	7 (50.0)	5 (35.7)	2 (14.3)	14 (51.9)

Almost all of the workers (96.3% [26]) who were injured and manifested symptoms due to an accident involving chemotherapy reported that at least one of the exposures had been by contact. Some reported that they were also exposed to aerosols 14.8% (4), ingestion 3.7% (1), or inhalation of neoplastic drugs 3.7% (1).

As can be observed from Table 3, showing the results for health care workers’ knowledge about biosafety, they were only partially informed. Of particular note among the eight questions, 30% (12) of the participants did not know what the main goal of chemotherapy treatment was and 52.5% (21) did not know how to proceed in case of an accident with antineoplastic drug material.

**Table 3:** Professionals' knowledge about chemotherapy, Porto Alegre, Brazil, 2014

Question	Correct n (%)	Incorrect n (%)
Primary purpose of treatment with antineoplastic drugs	28 (70.0)	12 (30.0)
Are there any regulations in Brazil regarding handling with antineoplastic drug?	32 (80.0)	8 (20.0)
Who is responsible for administration of chemotherapy?	33 (82.5)	7 (17.5)
What does "Work Accident Statement" mean?	25 (62.5)	15 (37.5)
Who is responsible for handling chemotherapy?	34 (85.0)	6 (15.0)
What precautions should be taken with excreta of patients on chemotherapy?	24 (60.0)	16 (40.0)
What are the main items of personal protect equipment?	29 (72.5)	11 (27.5)
How should you proceed in the event of an environmental accident	19 (47.5)	21 (52.5)

No significant statistical differences were detected in any of the tests using qualitative or quantitative variables.

## DISCUSSION

In this study, it was found that 90% (36) of the population surveyed were female. This result coincides with Lopes,^20^ who describes a process of feminization of health care workers, giving the female sex the role of “caring” as a biological and cultural origin claim on their gender.

It was observed that 35% (14) of participants were aged from 30 to 40 years. We know that antineoplastic drugs are teratogenic, meaning that they can cause birth defects and chromosomal aberrations (NIOSH^7^). It can therefore be concluded that most of the women surveyed are at a high risk to health because they are fertile. According to a literature review performed by Dranitsaris et al.,^21^ an association was identified between exposure to antineoplastic drugs and miscarriage. This is why there is still great concern with relation to these female health care workers.

It was shown in this study that 60% (27) of the population had suffered exposure related to antineoplastic drugs. Among the categories studied, pharmacists 40.74% (11) were the workers that most reported this experience, followed by nurses 33.33% (9), and technicians 25.93% (7). Due to the mutagenic and genotoxic potential of these medications, special care is required for all workers who handle these antineoplastic drugs, demanding technical and safety standards for handling of drugs and regular examinations of handlers.^6,7^

Regarding types of accident, 96.3% (26) of workers who reported having accidents had at least one accident by direct contact; 14.8% (4) reported inhalation of medications, 3.7% (1) ingestion, and 3.7% (1) inhalation exposure. During the observational period it became clear that the risk of an accident by skin contact with blood or chemotherapy was the most evident, because the health care workers were not wearing PPE adequately. This result coincides with studies published by Schreiber et al.^22^ and Black & Presson^23^ where the most common accidents involving professionals were by inhalation and skin contact, although involuntary ingestion through hand to mouth contact and unintentional injection by needle stick or sharp injuries were also possible.^22,23^ According to Hajjaji et al.,^24^ blood and body fluid exposure is a major occupational safety problem for health care workers and the increasing severity of blood exposure accidents is linked to the lack of safe behavior against this risk.^25^

The number of accidents in this study is underestimated, since only 44.4% (12) of the health care workers made a Work Accident Statement. There is also evidence of institutions making efforts to comply with legal requirements, since 92.5% (32) reported participating in the Program for Medical Control of Occupational Health and 72.5% (29) of the workers reported having some training in antineoplastic drugs. Itani^25^ stated that according to the Japanese Ministry of Health, Labor and Welfare, more than 1,000 workers are killed by occupational accidents and diseases every year, in Japan. He concluded that prevention of work-related diseases is essential to maintain and promote workers’ health and to ensure the quality of their working life.^24^ It is necessary that not only occupational health care staff but also clinicians pay greater attention to detection and prevention of work-related diseases.

The results for the health care workers’ knowledge about biosafety were quite surprising, since it was found that 30% (12) of the workers did not know the main goal of chemotherapy. This raises the following question: If health care workers who work with antineoplastic drugs do not even know the primary purpose of these drugs, how can one be sure they know how to protect themselves?

Recent research found that most health care workers are aware of the dangers of their work, but also receive limited training and a significant percentage of them do not wear PPE or wear it incorrectly.^26^ This corroborates our study showing that 72.5% (29) of the workers had been trained before starting these activities. However, we note that there was no record of this training in the facilities’ files.

Another important finding is that 52.5% (21) of the participants answered wrongly when asked how to proceed in an accident with antineoplastic drugs. This shows that, although all sites have spill kit in their units, they were not trained or had been trained wrongly to handle such an event. Combining these results with those of a study performed by Vollono et al.,^27^ it was found that most pharmacists (80%) and nurses (90.4%) had a high level of concern about dangerous antineoplastic drugs, that they do not have a satisfactory level of knowledge regarding risk factors, and that they do not have a high level of confidence in the safety measures adopted in the health care services to protect workers health. Kosgeroglu^28^ also found that nurses had quite a low level of information related to chemotherapy administration.

In conclusion, the present study aimed to identify occupational risks and evaluate the knowledge of health professionals who handle antineoplastic drugs and in order to achieve this these professionals were questioned and observed during their work activities.

Among health workers, nurses are part of the group most exposed to the various work activities that generate physical and mental stress. This does not refer only to the disease diagnosed, but also to loss of effective and/or potential, biological, and psychological capacity, which can compromise the worker’s ability to develop their biological and physical potential.

In relation to occupational risks, exposures by contact, aerosols, ingestion, and inhalation of neoplastic drugs were all identified. It is also known that ergonomic, physical, and biological risks are present, since working with different pathogenic organisms and work processes interferes in these workers’ health. Regarding the health professionals’ knowledge, there was a lack of information and weaknesses related to handling this type of medication.

With all of these findings, this study suggests there is a need to promote primary prevention, providing a safe environment for the employee, and to provide continuing education and training on safety measures. Furthermore, there is a need to develop and implement policies and guidelines on use of work practices and protective equipment. There should be greater oversight from regulatory authorities regarding the training received by these professionals. The health and safety of health care professionals is essential for treating cancer patients and treatment is most effective when they know that their own health is being preserved.
